# Exploring Factors of the Willingness to Accept AI-Assisted Learning Environments: An Empirical Investigation Based on the UTAUT Model and Perceived Risk Theory

**DOI:** 10.3389/fpsyg.2022.870777

**Published:** 2022-06-24

**Authors:** Wentao Wu, Ben Zhang, Shuting Li, Hehai Liu

**Affiliations:** College of Education Science, Anhui Normal University, Wuhu, China

**Keywords:** AI-assisted learning environment, college students, willingness to accept, UTAUT, perceived risk

## Abstract

Artificial intelligence (AI) technology has been widely applied in many fields. AI-assisted learning environments have been implemented in classrooms to facilitate the innovation of pedagogical models. However, college students' willingness to accept (WTA) AI-assisted learning environments has been ignored. Exploring the factors that influence college students' willingness to use AI can promote AI technology application in higher education. Based on the Unified Theory of Acceptance and Use of Technology (UTAUT) and the theory of perceived risk, this study identified six factors that influence students' willingness to use AI to analyze their relationships with WTA AI-assisted learning environments. A model including six hypotheses was constructed to test the factors affecting students' WTA. The results indicated that college students showed “weak rejection” of the construction of AI-assisted learning environments. Effort expectancy (EE), performance expectancy (PE), and social influence (SI) were all positively related to college students' WTA AI-assisted learning environments. Psychological risk (PR) significantly negatively influenced students' WTA. The findings of this study will be helpful for carrying out risk communication, which can promote the construction of AI-assisted learning environments.

## Introduction

Artificial intelligence (AI) technology is applied to promote learning and teaching effectiveness (Lim, 2020). AI technology is being used to monitor teaching processes and to perform academic analysis and academic level diagnosis (Zhai et al., 2021). Thus, reconstructing an AI-assisted learning environment with interactive and smart learning has become a new task in education. With AI-assisted learning environments, students' learning behavior and their interaction with teachers can be automatically recorded for further study (Yang et al., 2020). AI technology such as predictive modeling, intelligent analytics, assistive technology, automated content analysis, and image analysis applied in education can help solve important educational problems and ensure quality education (Salas-Pilco and Yang, 2022). The findings of previous studies can help promote the innovation of constructing a new learning environment with AI technology to improve the effectiveness of learning and teaching.

However, before constructing and implementing an AI-assisted learning environment, the learners' willingness to accept (WTA) should be considered. For learning in AI-assisted environments, studies on learners' attitudes toward AI technology are very important (Tahiru, 2021). However, students' real WTA classroom applications of AI have been ignored in education (Chai et al., 2020). Wang et al. (2020) proposed that AI technology applied in education should focus on the willingness of students, which is beneficial to make it effective. Lee (2019) also emphasized that the factors affecting students' willingness should be explored from the perspective of student-oriented learning. Thus, from the view of college students, the study aimed to examine the factors that affected their WTA AI-assisted learning environments. It is an important first step for the long-term effective application of AI technology in education.

Many theories and models have been applied in the studies on the acceptance of new technology (Al-Momani et al., 2019). Unified Theory of Acceptance and Use of Technology (UTAUT), proposed by Venkatesh et al. (2003), was considered the basic theory in the study. UTAUT model has been praised for predicting and interpreting users' behavioral intentions and behaviors regarding technology. The model has been applied in various studies of user behavioral intention in education (Uchenna and Oluchukwu, 2022). To measure students' WTA AI-assisted teaching environments, this study adopted the UTAUT model as it is one of the authoritative models used to investigate intention.

Unified Theory of Acceptance and Use of Technology includes four core dimensions, three of which point to willingness or perception, namely, performance expectation, social influence (SI), and effort expectation, and one of which points to use behavior, namely, facilitating conditions. Since an AI-assisted learning environment is a new technology-assisted learning environment, many students have no experience of learning in that kind of environment. The facilitating conditions which should be experienced with real use now can only be described to students. However, this study explored students' WTA AI-assisted learning environments. Therefore, we selected the three core dimensions as the variables in the UTAUT model that points to the willingness and explored their impact on students' willingness.

In the research on the acceptance of new technology, the theory of perceived risk is regarded as an important factor in the willingness to apply technology (Kim and Gu, 2012). As a new technology, Zhang et al. (2021) proposed to focus on the risks caused by the application of AI in education. To explore students' risk concerns about AI technology (Shin et al., 2017), we chose the perceived risk theory to comprehensively investigate the factors influencing students' WTA AI-assisted learning environments.

The concept of perceived risk was defined as “the risk of people predicting the outcome of the behavior before the behavior is implemented” (Bauer, 1960). It consists of six dimensions, namely, finance, function, physical, psychology, social, and time (Jacoby and Kaplan, 1972; Stone and Grønhaug, 1993). Combined with the characteristics of AI technology and based on the perceived risk theory, we selected the variables of functional, social, and psychological risk (PR) and removed the variables of financial, physical, and time risk to explore their impact on students' willingness.

Scholars have conducted a number of studies on AI technology, but there are few studies on the higher education application of AI technology. Additionally, existing studies have paid less attention to students' WTA AI-assisted learning environments. Thus, this study focused on students' WTA the construction and application of AI-assisted learning environments. It also explored the impact of six variables based on the UTAUT model and the perceived risk theory on students' willingness and proposed suggestions for the classroom application of AI technology. The study can provide theoretical implications for future study and the promotion of AI technology in higher education.

## Theoretical Background and Hypotheses

### AT Technology

According to Mata et al. (2018), AI technology is a broad scientific discipline that helps computer systems find ways to solve problems by simulating biological processes such as reasoning, learning, and self-correction. The theories, procedures, and technologies that help machines such as computers analyze, study, apply, and explore the essence of human thoughts and behaviors can be regarded as AI technology (Tan and Lim, 2018). It carries out data calculation through intelligent methods and applies the basic theories, methods, and technologies of computer hardware and software to simulate human behaviors, thus enabling computers to complete the tasks that only humans could complete in the past. Lu (2019) proposed that AI technology will simulate human interaction through their mother tongues, actions, and emotions in the future. Matsugu et al. (2003) found that AI technology may focus on the interaction between the human brain and machines in later research.

With the extensive application of AI, it has become deeply integrated into education. AI education has been transformed into educational AI (Lin et al., 2018). Intelligent teaching platforms, intelligent robots, and intelligent evaluation systems free teachers from tedious teaching and promote human-machine collaborative teaching (Luo, 2018). Weng et al. (2018) proposed that AI algorithms can be applied to financial courses to analyze the characteristics of the market economy. Tang et al. (2018) found that AI technology could be added to the radiology curriculum through a survey of students from Canada. However, few studies have taken into account the willingness of students to use AI technology before it is integrated into authentic educational contexts such as classrooms. There are also some academic studies on students' acceptance of AI-assisted learning. For example, Omer and Figen (2018) studied students' acceptance of mobile-assisted learning tools. Yuan et al. (2020) studied the acceptance of AI-assisted learning from the perspective of teachers. However, there has been less research on the construction of AI-assisted learning environments from the perspectives of students. Therefore, this study focused on students' views to explore their WTA the new learning environment.

### The UTAUT Model and Application in Education

When new technologies are applied, the users' acceptance should be first considered. Many models have been developed in previous studies for application in studies on the acceptance of new technology (Al-Momani et al., 2019). Specifically, the UTAUT model has succeeded in predicting and interpreting users' behavioral intentions and actual behaviors regarding technology. Additionally, the model has been applied in various studies of user behavioral intention in education (Uchenna and Oluchukwu, 2022). Based on Venkatesh et al.'s (2003) study, the UTAUT model was proposed combined with the technology acceptance model (TAM), the extended technology acceptance model (TAM2), and the theory of planned behavior (TPB), which has been shown to be a highly effective explanatory model (Wong et al., 2013; Oye et al., 2014). Scholars have achieved many findings in education based on the UTAUT model. Menant et al. (2021) further confirmed the validity of the TAM and UTAUT models on the basis of human resource information systems and users' acceptance. The UTAUT model has also been integrated with other theories, which aimed at establishing new models to solve specific problems (Chao, 2019). Existing studies have explored students' WTA new technology in education according to the UTAUT model (Li et al., 2020). Yakubu and Dasuki (2019) investigated higher education students in Nigeria based on the UTAUT model and found that the promotion conditions and behavioral intention were two significant factors influencing their actual use of educational technology. Ameri et al. (2020) conducted a survey on pharmaceutical students using a modified version of the UTAUT2 questionnaire, and the results indicated that performance expectancy (PE) and SI had positive effects on behavioral intention. Almaiah et al. (2019) applied the UTAUT model to explain students' acceptance of a mobile learning system in higher education and reported that perceived information quality and perceived security were the main factors of students' acceptance.

Four variables, namely, performance expectation, effort expectation, SI, and facilitating conditions, were in the UTAUT model. This study focused on students' willingness, which is a kind of perception, to accept the new learning environment, while not referring to the students' experience of the new environment, which is one kind of behavior. Many students have no experience of learning in this kind of environment. Thus, the variable of facilitating conditions was not considered in the study. We selected three core dimensions as the variables, namely, performance expectation, effort expectation, and SI, in the UTAUT model which point to willingness, and explored their impact on students' WTA AI-assisted learning environments.

#### Performance Expectancy

Performance expectancy (PE) is the positive impact that users perceive technology to have on their work (Venkatesh et al., 2003). A high adoption rate of technology means it works better. The UTAUT model was applied to investigate the intention to use PA apps among university students by Liu et al. (2019), and they found that PE positively affected the intention of PA apps usage. Li and Zhao (2021) conducted a study on the factors of continued intention to use MOOCs and found that PE had a positive effect on the intention. Studies have shown that performance positively influences students' WTA technology (Abbad, 2021). A hypothesis was thus proposed.

H1: There is a positive significant relationship between PE and students' WTA AI-assisted learning environments.

#### Effort Expectancy

Effort expectancy (EE) is the level of personal effort required to use technology (Venkatesh et al., 2003). According to existing studies, EE plays an indispensable role in the application of technology (Liebenberg et al., 2018). Altalhi (2021) conducted a survey of 150 students on MOOC acceptance and adaptability, and the data showed that effort expectations had a significant impact on student acceptance of MOOCs. When Yang et al. (2019) studied how the integrated model of UTAUT and Connected Classroom Climate (CCC) affected students' acceptance of cloud classrooms, they found that effort expectations had a significant effect. EE positively affects students' WTA the application of technology (Abbad, 2021). Thus, H2 was proposed.

H2: There is a positive significant relationship between EE and students' WTA AI-assisted learning environments.

#### Social Influence

Social influence means that when users use technology or service, they will continue to be influenced by the people and environment around them (Venkatesh et al., 2003). According to a study on college students' reception of social networking tools for learning in India, the results showed that the college students were influenced by SI which shaped their behavioral intentions (Alvi, 2021). According to an empirical study of electronic library service acceptance and the use of technology acceptance, the results showed that students' intention to use electronic library services depended on SI (Awwad and Al-Majali, 2015). When students use technology or services, their WTA will increase the willingness of their peers to use the technology or services (Ameri et al., 2020). Thus, we proposed the following hypothesis:

H3: There is a positive significant relationship between SI and students' WTA AI-assisted learning environments.

### Perceived Risk Theory

For studies on the acceptance of a certain new technology, users' perceived risk was regarded as an important factor affecting their willingness to use technology (Kim and Gu, 2012). Zhang et al. (2021) proposed that attention should be paid to the risks caused by the application of AI in education. Shin et al.'s (2017) study also focused on the exploration of students' risk concerns about AI technology. Thus, in this study, as a new learning environment for students, the perceived risk theory should be considered in the factors influencing students' WTA AI-assisted learning environments.

Bauer (1960) first proposed the theory of perceived risk and defined it as “the risk of people predicting the outcome of the behavior before the behavior is implemented.” Jacoby and Kaplan (1972) proposed that it consisted of five dimensions, namely, finance, function, body, psychology, and society. Based on the theory of perceived risk, Stone and Grønhaug (1993) increased the dimension of time risk through an experimental exploration. Venkatesh et al. (2003) developed four core variables that have a significant impact on age, experience, gender, and voluntariness. They further proposed that the compound effect of more than two variables would produce a more significant influence. Lăzăroiu et al. (2020) analyzed the decision-making process of consumers from three dimensions of perceived risk. Commodari and La Rosa (2020) studied the body risks, PRs, beliefs, and expectations of quarantined young people in Italy during COVID-19. However, few studies in higher education have focused on the theory of perceived risk.

Combining the specific issues to be explored, we selected the three independent variables of function, society, and psychology from the theory of perceived risk due to the following reasons: (1) the main feature of AI technology application in college classrooms is high efficiency (Lukas et al., 2016). (2) The classroom application of AI technology will not have an impact on the health of college students and will avoid causing physical risks (Lin, 2000). (3) The classroom application of AI technology generally does not involve economic behavior and avoids causing financial risks.

The theory of perceived risk has been incorporated into the decision hypothesis model and widely applied in the studies of technology acceptance willingness (Kim and Gu, 2012; Kim, 2019). Based on the functional characteristics of AI technology (Xia et al., 2018; Hoo and Ibrahim, 2019; Yang and Han, 2020; Zhu et al., 2020), college students may face functional risk (FR), PR, and social risk (SR) when accepting AI technology in the classroom. The risks negatively impact their WTA face recognition technology (Wei et al., 2021). Thus, the following hypotheses were proposed:

H4: There is a negative significant relationship between FR and students' WTA AI-assisted learning environments.
H5: There is a negative significant relationship between PR and students' WTA AI-assisted learning environments.
H6: There is a negative significant relationship between SR and students' WTA AI-assisted learning environments.

**Figure 1:**
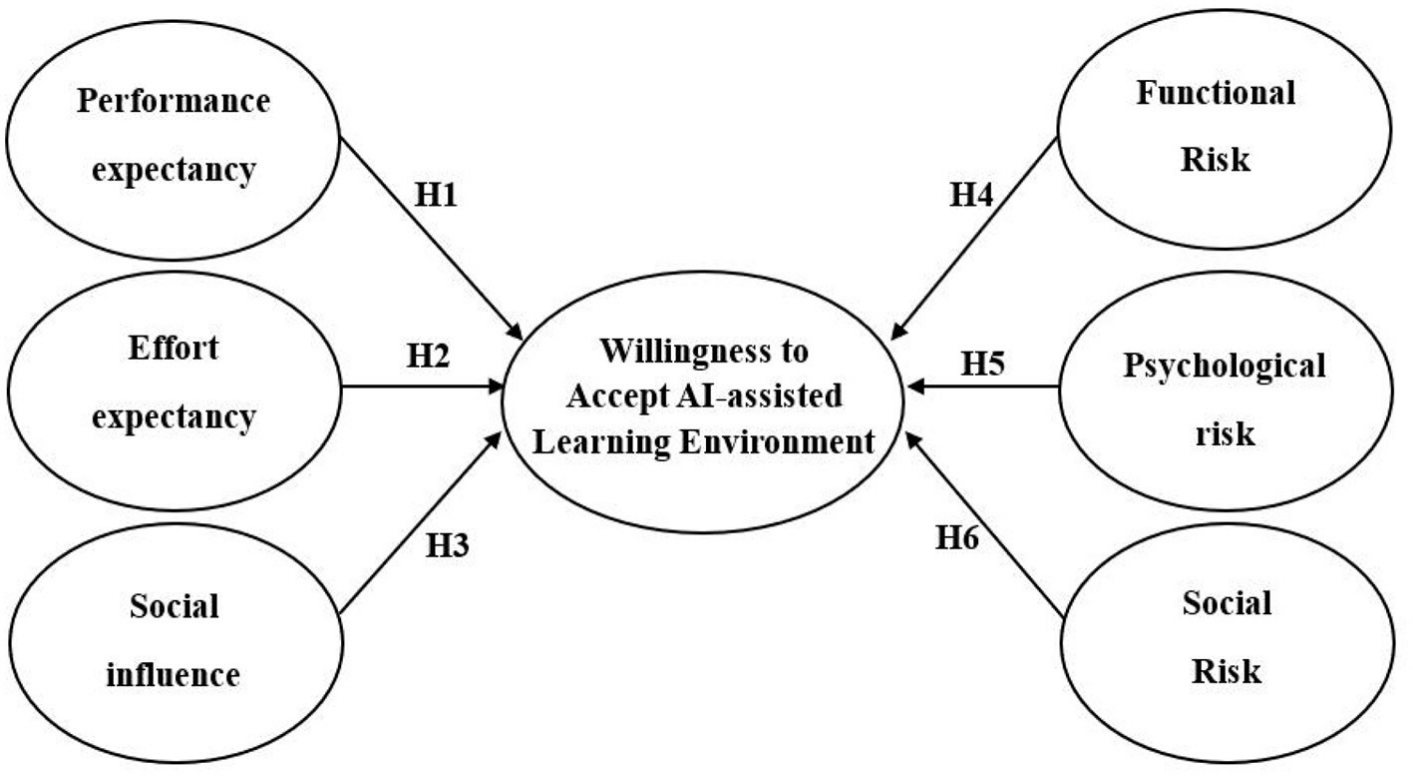
Research model.

The model is illustrated in Figure 1.

## Methods

### Participants

The participants were college students from four universities in Anhui Province, China, which were planning to construct AI-assisted learning environments. A questionnaire was developed to be sent to an online survey website named Questionnaire Star (www.wjx.cn), an online survey tool commonly used in many studies (Liu et al., 2022; Zhu et al., 2022). It supports easy access for senders and receivers *via* a web address. With the help of teachers from the four universities, the address of the online questionnaire was sent to the students. The first part of the questionnaire was the informed consent letter. Students were informed that the questionnaire was anonymous and would not have any impact on their daily life or academic studies. This survey was completely voluntary. If the participants knew and agreed with the instructions, they would start answering the questions, which meant that they also agreed to participate in this survey; if they were unwilling to participate, they could not answer the questions and exited the web page. Finally, 2,238 online questionnaires were obtained. Those questionnaires with the same answers and with responses given within an overly short answering time were deleted, leaving 1,655 valid questionnaires, with a 73.95% recovery rate. As shown in Table 1, female college students accounted for the most respondents, comprising 65.86% (1,090), with male college students accounting for 34.14% (565). Among these students, those majoring in the liberal arts accounted for the most, at 52.51% (869), while science students accounted for 47.49% (786).

**Table 1:** The specific composition of the sample.

**Demographic variables**	**Types**	***N***	**Percentage**	**Mean**	**Std. deviation**
Gender	Male	565	34.14%	2.90	0.942
	Female	1,090	65.86%	2.80	0.984
Subject	Liberal arts	869	52.51%	2.78	1.023
	Science	786	47.49%	2.94	0.946
Total.		1,655		2.84	1.102

### Instrument

A questionnaire was designed for data collection. The questionnaire comprised three parts; the first part was the explanatory information, the second part was the survey of demographic information, and the third part was the scale of seven variables, namely, PE (three items), SI (three items), EE (three items), FRs (three items), SR (three items), PR (three items), and students' WTA (three items). Based on previous scales, the “Students' WTA AI-Assisted Learning Environments Questionnaire” was developed using a 5-point Likert scale. The survey was conducted in two steps. First, 40 college students were invited to participate in the pre-survey to test the validity and reliability of the questionnaire. Combined with the test results, the questionnaire was revised. Then, it was distributed online.

#### Performance Expectancy

Three items were used to measure PE to understand students' perceived effect of AI-assisted teaching environments on their learning process. This study adopted the PE scale developed by Venkatesh et al. (2003) and Chatterjee and Bhattacharjee (2020) to reflect students' perceptions of AI technology improving the learning process. Sample items are “I think AI technology can increase my attention to this class” and “I think AI technology can improve my learning attitude.”

#### Effort Expectancy

Three items were used to measure EE to understand student use of AI technology. This study adopted the EE scale developed by Venkatesh et al. (2003) and Yang et al. (2019) to reflect the difficulty level of AI technology application by students. Sample items are “I think the AI technology behavior analysis system is easy to learn” and “I think the information fed back by AI technology in the classroom is easy to understand.”

#### Social Influence

Three items were used to measure SI to understand the impact of the external environment on student learning using AI technology. This study adopted the SI scale developed by Venkatesh et al. (2003) and Awwad and Al-Majali (2015) to reflect that students' use of AI being affected by the external environment. Sample items are “I can get publicity about the AI-Assisted Learning Environment from school” and “There are classmates and teachers around me who suggest using the AI-Assisted Learning Environment.”

#### Functional Risk

This scale was adapted from Jacoby and Kaplan (1972) and Kim and Gu (2012) to address students' deficiencies in AI technology function. Three items were designed to assess students' FR using AI technology. Sample items include “I am worried that AI technology will feed back wrong information about classroom activities” and “I am worried that some functions of AI technology in the classroom will not be available and will cause trouble.”

#### Social Risk

This scale was adapted from Jacoby and Kaplan (1972) and Chen et al. (2021) to address social pressures on students' use of AI technology. Three items were designed to assess students' SR from AI technology. Sample items include “I'm worried that using AI technology will lead to a bad evaluation from my teachers” and “I am worried that the online data of the AI-Assisted Learning Environment will expose me to invisible supervision.”

#### Psychological Risk

This scale was adapted from Jacoby and Kaplan (1972) and Chen et al. (2021) to address the physical and mental stress of students using AI technology. Three items were designed to assess students' PR from AI technology. Sample items include “The AI-Assisted Learning Environment will make me afraid of classes like this” and “The AI-Assisted Learning Environment makes me feel that I am not trusted.”

#### Students' Willingness to Accept

Three items were used to measure WTA to understand students' receptive attitudes toward AI technology. This study adopted the WTA scale developed by Venkatesh et al. (2003) and Chatterjee and Bhattacharjee (2020) to reflect students' perceptions of AI technology acceptance. Sample items are “In the future, I am willing to use AI-Assisted Learning Environments” and “In the future, I will recommend that others use AI-Assisted Learning Environments.”

### Validity and Reliability of Instruments

The validity and reliability were tested by Cronbach's alpha coefficient analysis, composite reliability (CR) analysis, and factor loading coefficient analysis (refer to Table 2). The factor loading coefficients of items are more than 0.5, indicating the excellent reliability of items in this questionnaire. The CR values are more than 0.65, and the alpha coefficient values are more than 0.7, indicating that the variables involved in the item can be consistent. Explaining the latent variable in an appropriate manner has excellent combination reliability (reflecting internal consistency). The formula used to calculate CR is Σλ2/(Σλ2+Σε). The AVE values of the measured variables are more than 0.5, indicating excellent internal consistency validity of the questionnaire. The formula used to calculate AVE is Σλ2/*n*. Thus, the questionnaire shows high reliability and validity, and the reliability of the collected data is excellent.

**Table 2:** Reliability and validity of instruments.

**Main variable**	**Load factor of** **each item factor**	**AVE**	**CR**	**Alpha**
PE	0.805–0.959	0.8177	0.9305	0.926
EE	0.651–0.978	0.7367	0.8909	0.874
SI	0.839–0.903	0.7699	0.9093	0.909
FR	0.761–0.921	0.7561	0.9023	0.900
PR	0.851–0.908	0.7898	0.9185	0.918
SR	0.895–0.976	0.8873	0.9593	0.959
WTA	0.841–0.881	0.7445	0.8973	0.915

*WTA, willingness to accept; PE, performance expectancy; SI, social influence; EE, effort expectancy; FR, functional risk; PR, psychology risk; SR, social risk*.

### Data Analysis

In this study, first, SPSS24.0 was applied to test the validity and reliability of the scale. Then, the correlation analysis between six variables and WTA was conducted. AMOS 24.0 was utilized to analyze the structural equation model (SEM). SEM was used to test the reliability and validity of the model and to calculate the path coefficient, so as to obtain valuable conclusions.

## Results

### Analysis of Correlation of Six Variables With the WTA

To explore the correlation among the variables related to college students' WTA AI-assisted learning environments, this study adopted Pearson product-moment correlation analysis to analyze the relationship among variables. The results show that there was a significant correlation between WTA and the other six variables (refer to Table 3).

**Table 3:** Correlation coefficient among core variables (*N* = 1,655).

	**WTA**	**PE**	**EE**	**SI**	**FR**	**PR**	**SR**
WTA	1.000						
PE	0.600**	1.000					
EE	0.357**	0.246**	1.000				
SI	0.481**	0.338**	0.050*	1.000			
FR	−0.281**	−0.104**	−0.133**	−0.186**	1.000		
PR	−0.229**	−0.103**	−0.175**	−0.053**	0.194**	1.000	
SR	−0.049**	0.005	−0.233**	−0.239**	0.050*	−0.237**	1.000

*p < 0.05, **p < 0.01; *means there is a significant correlation at the 0.05 level. **means there is a significant correlation at the 0.01 level.

### Modification and Verification of the Model

To verify the interpretation degree of the model, AMOS 24.0 was used to evaluate the SEM. The evaluation standard coefficient was compared to indicate that the four fitting indexes of the model were all within the acceptable range (refer to Table 4). Therefore, the structure of the modified model was reasonable.

**Table 4:** Model fitting results of influencing factors of college students' acceptance intention after modification.

**The index name**	**The evaluation index**		**The actual index**
	**Well**	**Acceptable**	
GFI (Goodness of fit index)	>0.9	0.7–0.9	0.923
AGFI (Adjusted goodness of fit index)	>0.9	0.7–0.9	0.903
CFI (Goodness-of-fit index)	>0.9	0.7–0.9	0.959
RMESA (Root Mean Square Residual)	<0.08	0.08–0.1	0.063

As Table 5 shows, the paths among variables in the revised model were all significant, indicating that research hypotheses H1, H2, H3 H4, H5, and H6 were all supported.

**Table 5:** Path analysis results of the model influencing factors.

**Path**	**Estimate**	**S.E**.	**C.R**.	***p***	**Standardized estimate**	**Hypothesis**
WTA ← PE	0.468	0.021	22.238	0.000	0.516	H1 was supported.
WTA ← EE	0.297	0.026	11.559	0.000	0.233	H2 was supported.
WTA ← SI	0.326	0.020	16.721	0.000	0.364	H3 was supported.
WTA ← FR	−0.153	0.020	7.744	0.000	−0.158	H4 was supported.
WTA ← PR	−0.182	0.020	9.185	0.000	−0.188	H5 was supported.
WTA ← SR	−0.090	0.019	4.618	0.000	−0.091	H6 was supported.

## Discussion

### PE, SI, and EE Had a Positive Significant Relationship With College Students' WTA AI-Assisted Learning Environments

Previous studies have found that, with the improvement of PE, SI, EE, and facilitating conditions (Awwad and Al-Majali, 2015; Altalhi, 2021; Alvi, 2021), the willingness of college students to accept AI-assisted learning environments increased significantly; the same result was found in this study.

Performance expectancy had a positive significant relationship with students' WTA AI-assisted learning environments, and its direct impact load was 0.468, which was the most significant of all the variables. H1 was supported. This finding was consistent with Li and Zhao's (2021) study. Students believe that AI-assisted learning environments can improve their learning attitude, optimize their learning, and improve their learning efficiency (Lai, 2021). It can be seen that students pay more attention to the help of the AI-assisted learning environment for their learning. Thus, improving the learning effect is the primary purpose when constructing and implementing AI-assisted learning environments.

Effort expectancy had a positive significant relationship with students' WTA AI-assisted learning environments, and its direct impact load was 0.297. H2 was supported. Liebenberg et al. (2018) found that PE, SI, and EE had a moderate linear positive influence on college students' acceptance intention. The difficulty of technology application has a negative impact on students' use of AI-assisted learning environments. When students think that the use method of technology is easy to learn and the data fed back by technology is easy to understand, they will be more receptive to AI-assisted learning environments (Akgun and Greenhow, 2021). Therefore, constructing and implementing AI-assisted learning environments need to take into account students' information technology levels.

The social influence had a positive significant relationship with students' WTA AI-assisted learning environments, and its direct impact load was 0.326. H3 was supported. If college students do not know enough about AI-assisted learning environments or have insufficient experience, their WTA AI-assisted learning environments will be weakened. Therefore, SI is an important factor in improving students' attitudes toward AI-assisted learning environments.

### FR, PR, and SR Had a Negative Significant Relationship With College Students' WTA AI-Assisted Learning Environments

Functional risk had a negative significant relationship with students' WTA AI-assisted learning environments, and its direct impact load was −0.153. H4 was supported. Kim and Park (2020) proposed that AI-assisted learning environments rely on technology. Due to the limitations of technology, there will be inaccurate learning data. AI-assisted learning environments may produce inaccurate learning information due to algorithm errors (Shin, 2019). Thus, students' concerns about the technical function of AI-assisted learning environments will have a negative impact on their WTA such environments.

Psychology risk (PR) had a negative significant relationship with students' WTA AI-assisted learning environments, and its direct impact load was −0.182. H5 was supported. This finding was similar to Khan and Khan's (2019) study. Compared with the traditional learning environment, the new one will have an impact on students' psychological state, which can make students experience psychological pressure and become very nervous. These factors have a negative impact on students' acceptance of AI-assisted learning environments.

Social risk had a negative significant relationship with students' WTA AI-assisted learning environments, and its direct impact load was −0.090. H6 was supported. Wang (2021) proposed that the mistakes of AI technology in data security and privacy will have a negative impact on learners. In the AI-assisted learning environment, all kinds of data generated during the learning process are recorded, which may generate some negatively evaluated information. Students may face SRs caused by external evaluation. Students' concern about potential SRs has a negative impact on their acceptance of AI-assisted learning environments.

## Conclusion

In this study, PE, SI, and PE had a positive significant relationship with college students' WTA AI-assisted learning environments. The most important factor influencing students' WTA AI-assisted learning environments was EE, while FR, PR, and SR also had a negative significant relationship with college students' WTA AI-assisted learning environments.

### Implications

Theoretically, the findings of this study can provide implications for designers and teachers of AI-assisted learning environments. First, in this study, a new hypothetical model was constructed based on the UTAUT model and the perceived risk theory for the first time and successfully verified the effectiveness of the factors affecting students' WTA AI-assisted learning environments. Second, it was found that the core variables of UTAUT have a positive impact on students' WTA AI-assisted learning environments. Thus, the designers and teachers who design and apply AI-assisted learning environments should focus on students' PE, EE, and SI. With improvement in these factors, students' acceptance willingness increased significantly. Third, key variables in the perceived risk theory negatively influence students' WTA AI-assisted learning environments. Therefore, it is necessary for designers and teachers to provide help when students use AI technology, which can reduce students' worries and increase their WTA. Thus, a platform for communication should be built to pay attention to students' needs and opinions. An appropriate risk communication mechanism should be established to carry out risk communication at different stages of technology application, so as to solve the potential problems college students are worried about.

As for the practical value of this study, it aims to promote the application of AI technology in education and the construction of AI-assisted learning environments. During the whole application of AI-assisted learning environments, students should be informed of the positive purpose of technology application before use, and targeted improvements should be made based on students' opinions and attitudes. Teachers should explain the reasonable way of technology application to alleviate students' anxiety when using AI technology in the instruction. After use, students' psychological influence should be studied to avoid the negative impact on students' mental and physical health. Therefore, the purpose of the above measures is to make students feel respected throughout the whole process of technology application, so as to eliminate the PR caused by the crisis of trust and to improve their WTA the new environment.

Additionally, for following studies or for the application of new learning environments, it is suggested that enriching the function of AI technology and enhancing the effectiveness of education are necessary. AI-assisted learning environments can be optimized to provide more comprehensive and accurate analysis and feedback in the classroom. AI technology in the classroom should be further developed to a larger scale of applications, such as class attendance records, learning behavior analysis, teaching behavior analysis, teachers' and students' interaction analysis, emotion calculation, and humanistic care research, which is favorable for deep integration of AI-assisted learning environments and classroom instruction.

### Limitations and Future Studies

Although this study enriches the relevant theories of the classroom application of AI, there are still some defects to be improved in future research. First, the sample did not cover all college students. In future studies, more extensive and representative samples should be collected to verify the conclusions of this study. Second, this study established influencing factors on students' willingness based on the UTAUT model and perceived risk theory, ignoring other variables such as gender and age, which should be considered in future studies.

## Data Availability Statement

The original contributions presented in the study are included in the article/supplementary material, further inquiries can be directed to the corresponding author/s.

## Ethics Statement

Ethical review and approval were not required for the study on human participants in accordance with the local legislation and institutional requirements. Written informed consent for participation was not required for this study in accordance with the national legislation and the institutional requirements.

## Author Contributions

All authors contributed equally to the conception of the idea, implementing and analyzing the experimental results, writing the manuscript, and reading and approving the final manuscript.

## Funding

This study was funded by the Anhui Philosophy and Social Science Planning Project (Youth Project) Research on Influencing Factors of Application Policy Implementation Effect of Smart Schools in Anhui Province (AHSKQ2021D43) in 2021.

## Conflict of Interest

The authors declare that the research was conducted in the absence of any commercial or financial relationships that could be construed as a potential conflict of interest.

## Publisher's Note

All claims expressed in this article are solely those of the authors and do not necessarily represent those of their affiliated organizations, or those of the publisher, the editors and the reviewers. Any product that may be evaluated in this article, or claim that may be made by its manufacturer, is not guaranteed or endorsed by the publisher.
